# Cerebro-Spinal Meningitis

**Published:** 1907-02-16

**Authors:** 


### The Hospital

A Journal of

The Medical Sciences and Hospital Administration.

Vol. XLI.—No. 1066. SATURDAY, FEBRUARY 16, 1907.

### Cerebro-Spinal Meningitis.

The reports which are being received from various large towns, and particularly from Glasgow and Belfast, afford convincing evidence of the fact that cerebro-spinal meningitis in an epidemic form has managed to gain a foothold in this country. Hitherto it has been a somewhat curious feature in the natural history of the disease that, while occasional cases, and even one or two localised outbreaks, have been reported in Great Britain, there have never been any such extensive epidemic developments as have marked the course of the malady in America and Central Europe. In the absence of any adequate explanation of this apparent immunity, it has long been obvious that no sufficient guarantee of its continuation existed. In view of this, and of the outbreaks of the disease reported from various countries in the course of last year, the Local Government Board was well within its duty in directing the attention of the local health authorities in England and Scotland to the possibilities of epidemics appearing in this country. When commenting on the Board's circular, immediately after its issue, we ventured to urge that a policy of compulsory notification should at once be enforced. That suggestion has been abundantly justified by the subsequent facts, and in large centres, such as Glasgow, where it has been adopted, a considerable body of information has now been collected. This information, unfortunately, does not so far point decisively to any method by which the disease can be either cured or prevented. But, as many experiences have shown, it is only by the systematised collection and study of numerous cases of any disease that the true nature and natural history of the disease can be appreciated, and such appreciation is an essential preliminary to the promotion of the practical measures necessary to protect both the community and the individual. Hence we once again venture to press on all local health authorities their responsibility in this matter, and to suggest that the provisions of the Infectious Diseases (Notification) Act should be immediately applied to cerebro-spinal meningitis in all parts of the country. The official circular issued by the Local Government Board conveyed an intimation that the Board would be prepared to consider applications in this direction in "special circumstances." With such reports as are coming from the Northern towns we think it may be considered that this condition is fully satisfied, and hence we hope that without further delay compulsory notification will be universally enforced.

It has been suggested that the explanation of the more frequent records of cases of cerebro-spinal fever is to be found, not in a greater prevalence of the disease, but in its more ready recognition as a result of improved methods of diagnosis. This question is discussed in a report recently issued by Dr. Chalmers, Medical Officer of Health for Glasgow, and the facts submitted fully justify, at least in our opinion, the conclusion that such an explanation of the present outbreak is not tenable. Indeed, it may be questioned whether the true proportions of the epidemic are not to some extent concealed by the failure in individual instances to distinguish cerebro-spinal fever from other forms of meningitis. Thus the Glasgow records show, that in 1906, and especially in the latter half of the year, there was a mysterious and gradual increase in the number of deaths certified as due to tubercular meningitis. During the previous five years the deaths from that disease per annum ranged from 237 to 260, with an average of 244; in 1906 the figures were 307. It is impossible to resist the suspicion that some of this increase is probably to be explained by a failure to recognise individual instances of the cerebro-spinal variety of meningitis. And the suspicion is made still stronger when it is announced that the Glasgow returns for 1906 actually include 147 cases of this last-mentioned malady; while in January 1907, 162 further cases were certified. No doubt some of these were proved to be mistaken diagnoses, but such errors are probably fully compensated for by the cases of cerebro-spinal meningitis which were confounded with and registered as examples of other diseases. In regard to this question of diagnosis attention may once more be directed to the value of lumbar puncture. It is easily performed, and, with proper precautions, is entirely safe. What its therapeutic position may be is a matter of uncertainty, but by allowing bacteriological examination of the cerebro-spinal fluid it affords an opportunity for a final and definite diagnosis, though there is reason to believe that a negative observation is by no means decisive.

From the outbreak at Glasgow it is not, unfortunately, possible to gather much light regarding the method by which infection is spread. Multiple cases in one and the same household have been repeatedly noted, but they have mostly been so closely related in date of onset as to suggest simultaneous infection. A considerable number of cases have occurred among breast-fed infants who, so far as could be traced, had no association with patients suffering from the disease. Dr. Chalmers suggests a possible relationship between the epidemic and the recent low temperatures, which, by lessening vitality, may, it is suggested, diminish the effective resistance of the tissues to the specific germ. Whether anything more definite emerges from this suggestion or not, it is all to the good that the policy of compulsory notification adopted at Glasgow has brought the disease into the sphere of those who are most competent to study its manifestations and to control its incidence.

